# Fluorescent peptide dH3w: A sensor for environmental monitoring of mercury (II)

**DOI:** 10.1371/journal.pone.0204164

**Published:** 2018-10-10

**Authors:** Marialuisa Siepi, Rosario Oliva, Luigi Petraccone, Pompea Del Vecchio, Ezio Ricca, Rachele Isticato, Mariamichela Lanzilli, Ornella Maglio, Angela Lombardi, Linda Leone, Eugenio Notomista, Giuliana Donadio

**Affiliations:** 1 Department of Biology University of Naples Federico II, Naples, Italy; 2 Department of Chemical Sciences University of Naples Federico II, Naples, Italy; 3 IBB, CNR, Naples, Italy; Chinese Academy of Sciences, CHINA

## Abstract

Heavy metals are hazardous environmental contaminants, often highly toxic even at extremely low concentrations. Monitoring their presence in environmental samples is an important but complex task that has attracted the attention of many research groups. We have previously developed a fluorescent peptidyl sensor, dH3w, for monitoring Zn^2+^ in living cells. This probe, designed on the base on the internal repeats of the human histidine rich glycoprotein, shows a turn on response to Zn^2+^ and a turn off response to Cu^2+^. Other heavy metals (Mn^2+^, Fe^2+^, Ni^2+^, Co^2+^, Pb^2+^ and Cd^2+^) do not interfere with the detection of Zn^2+^ and Cu^2+^. Here we report that dH3w has an affinity for Hg^2+^ considerably higher than that for Zn^2+^ or Cu^2+^, therefore the strong fluorescence of the Zn^2+^/dH3w complex is quenched when it is exposed to aqueous solutions of Hg^2+^, allowing the detection of sub-micromolar levels of Hg^2+^. Fluorescence of the Zn^2+^/dH3w complex is also quenched by Cu^2+^ whereas other heavy metals (Mn^2+^, Fe^2+^, Ni^2+^, Co^2+^, Cd^2+^, Pb^2+^, Sn^2+^ and Cr^3+^) have no effect. The high affinity and selectivity suggest that dH3w and the Zn^2+^/dH3w complex are suited as fluorescent sensor for the detection of Hg^2+^ and Cu^2+^ in environmental as well as biological samples.

## 1. Introduction

Heavy metal ions when present in excess are toxic for all organisms [1]. They are difficult to remove from the environment and unlike many other pollutants cannot be chemically or biologically degraded, therefore, heavy metals constitute a global environmental hazard [2]. Mercury and its compounds, in particular, are regarded as “priority hazardous substances” by the Agency for Toxic Substances and Disease Registry (ATSDR) because of its toxicity, mobility, long residence time, and biomagnification in food chains [3]. For examples, Hg^2+^ is highly toxic even at low levels [4] and in humans it can affect liver, kidneys and the cardiovascular, gastro-intestinal and neurological systems [5]. Hg^2+^ detection is undoubtedly significant in environment and health monitoring. This has prompted the development of highly selective and sensitive, low cost chemosensors for the detection of Hg^2+^ in environmental and biological samples. Many of these Hg^2+^ sensors are based on colorimetric and/or fluorometric detection [6–13], but very sensitive sensors based on surface enhanced Raman spectroscopy (SERS) [14], chiroptical signal and electrical conductivity [15] have also been developed.

Fluorescent sensors usually have a metal binding module (often an organic ligand) linked to a fluorescent moiety [6–13]. The binding of a specific metal ion to the sensor, through mechanisms like photoinduced electron transfer (PET) and chelation enhanced fluorescence (CHEF), causes variation in the fluorescence thus allowing an easy and real-time detection of the metal/sensor interaction [7]. However, some colorimetric and fluorometric sensors exploit metal catalyzed reactions like spirocyclic ring opening in rhodamine derivatives [7] or deprotection of dithioacetals ([8] and references therein). Major drawbacks of these sensors can be difficult synthesis, limited solubility in water or the fact that they undergo an irreversible reaction with the analyte.

Metal binding proteins are intriguing alternatives to organic ligands. Protein-based Hg biosensor based on electrochemical [16,17] and optical [18] techniques has been already prepared. However, poor stability of proteins at ambient conditions limit their applications. On the other hand, short metal binding peptides are particularly suited for the development of fluorescent probes. The structural complexity and the flexibility of peptides allow to exploit metal induced conformational changes as part of the sensing mechanism [19], moreover, peptidyl probes properties can be tuned by changing/mutating specific amino acids as well as changing the fluorescent moiety and the residue to which it is attached. In addition, short peptides can be easily synthesized via 9-fluorenyl-ethoxycarbonyl (Fmoc) solid-phase peptide synthesis (SPPS) [20], and can be generally employed in aqueous solutions. In previous work [19] we devoted our attention to histidine-rich glycoproteins (HRGs), complex multidomain proteins found in the serum of vertebrates, characterized by an impressive variety of functions in blood coagulation, fibrinolysis and in the innate immune systems [21]. We demonstrated that the designed peptide dH3w (dansyl-HPHGHW-NH_2_), containing the repeated sequence of human HRG (HPHGH) plus a dansyl group and a tryptophan residue at the two ends, can be used as a turn-on fluorescent probe for Zn^2+^ and a turn-off probe for Cu^2+^ in biological systems [19]. Here we demonstrate that dH3w has an affinity for mercury (II) ions 1000 times higher than that for Zn^2+^, thus being able to displace zinc from the dH3w/Zn^2+^. With the exception of Cu^2+^ no other heavy metal is able to displace Zn^2+^, therefore, dH3w complex with Zn^2+^ could be used as selective probes for environmental monitoring of Hg^2+^ and Cu^2+^.

## 2. Materials and methods

### 2.1 Chemicals

All chemicals were reagent grade unless noted, and deionized distilled water was used to prepare solutions. Peptide dH3w (dansyl-His-Pro-His-Gly-His-Trp-NH_2_) was synthesized by Primm srl. with a purity grade of 98% (S1 and S2 Figs). Stock solutions of metal ions were prepared by dissolving the appropriate chloride salt in a 20 mM Mops [3-(N-morpholino) propanesulfonic acid] buffer, pH 7.0. All samples solutions were prepared by proper dilution of stock solutions.

### 2.2 Steady-state fluorescence measurements

Steady-state fluorescence spectra were recorded on a Fluoromax-4 fluorometer (Horiba, Edison, NJ, USA) using a 1 cm path length quartz cuvette. The temperature was set to 25 °C. The excitation wavelength was set to 340 nm and emission spectra were recorded in the range 400 nm– 660 nm. The slit widths for excitation and emission were set to 2 nm and 3 nm, respectively. Fluorescence emission spectra as a function of Hg^2+^ concentration were recorded by titrating a solution of dH3w (7 μM) with a solution of Hg^2+^. All the experiments were carried out in 20 mM Mops buffer adjusted at the appropriate pH values with the addition of sulfuric acid or sodium hydroxide. For displacement experiments, a solution of dH3w (7 μM) saturated with Zn^2+^ (100 μM), was titrated with a solution of Hg^2+^ ranging from 0 up to ~ 100 μM. The fraction of bound peptide (α) at each point of titration was obtained by following the changes in the fluorescence intensity at the maximum emission wavelength by means of the equation:

$$\alpha =\frac{I_{\lambda }-I_{\lambda }^{free}}{I_{\lambda }^{bound}-I_{\lambda }^{free}}$$

where *I*_*λ*_ is the fluorescence intensity at each point of the titration, $I_{\lambda }^{free}$ is the intensity of free peptide and $I_{\lambda }^{bound}$ is the intensity of the peptide at saturation. The displacement binding curve was obtained by plotting the fraction of bound peptide (α) versus Hg^2+^ concentration and the experimental points were fitted using a two non-equivalent and independent binding sites model.

### 2.3 Metal selectivity studies

dH3w (10 μM) and Zn^2+^ (100 μM) were incubated in the presence of different concentrations (30, 60 and 90 μM) of metal ions (Cu^2+^, Ni^2+^, Co^2+^, Mn^2+^, Pb^2+^, Cd^2+^, Fe^2+^, Sn^2+^ and Cr^3+^) and the fluorescence intensity at 515 nm was recorded after excitation at 340 nm, using a plate reader (Synergy HTX Multi-Mode Reader-BIOTEK).

### 2.4 Job’s plot

To check all the possible binding stoichiometries between dH3w and Hg^2+^ ions, the continuous variations method (Job’s plot) was applied [22]. The mole fraction of dH3w ranged from 0.1 to 0.9 and the total molar concentration (dH3w+Hg^2+^) was fixed at 40 μM.

### 2.5 Quantum yield determination

Fluorescence quantum yields in the presence of Hg^2+^ (100 μM) were determined as previously described [19] using fluorescein as a standard. Measurements were performed in 20 mM Mops buffer, pH 7.0. All emission spectra were recorded in the range 360–700 nm after excitation at 350 nm.

### 2.6 Determination of the detection limit

The detection limit (LOD) for Hg^2+^ was calculated by performing a fluorescence titration. The emission intensity of free dH3w was measured 10 times and the standard deviation determined. Then, calibration curves were obtained by recording the fluorescence at Hg^2+^ concentrations up to 1200 nM. Each calibration curve was obtained in triplicate. The detection limit was then calculated as LOD = 3σ/k, where σ is the standard deviation of the free peptide measurements and k is the slope of the line obtained by plotting fluorescence intensity versus the metal ion concentration [23]. The detection limit was calculated at pH 4.0 and 7.0.

### 2.7 NMR spectroscopy

All NMR spectra were acquired at 298 K on Bruker Avance 600 MHz spectrometer, equipped with triple resonance cryo-probe. NMR characterization was performed in H2O/D_2_O (90/10 v/v).

NMR samples of dH3w were prepared by dissolving weighted amounts of the peptide in the solvent systems (V = 0.520 μl) for a final concentration of 0.5 mM. Metal/peptide complex was prepared using the following conditions: to a 0.5 mM dH3w solution in 90% H_2_O/10% D_2_O (pH 3.8), small aliquots (2.6 μL) of a freshly prepared aqueous stock solution of HgCl_2_ (25 mM) were added until a 1:1 peptide:metal ratio was reached. The pH was then adjusted to 4.5 with NaOH and the concentration of Hg^2+^ was gradually increased up to 10 mM.

Chemical shifts were referenced to internal sodium 3-(trimethylsilyl)[2,2,3,3-d_4_] propionate (TSP). Two-dimensional (2D) experiments, such as total correlation spectroscopy (TOCSY)[24], nuclear Overhauser effect spectroscopy (NOESY) [25,26], rotating frame Overhauser effect spectroscopy (ROESY) [27], double quantum-filtered correlated spectroscopy (DQF-COSY) [28] and Heteronuclear single quantum coherence (1H, 13C HSQC) were carried out with standard pulse sequences. The data file generally consisted of 512 and 2048 (4096 for DQF-COSY) data points in the ω1 and ω2 dimensions, respectively, which was zero-filled to obtain 2048 x 4096 data points in the spectrum. In all homonuclear experiments the data matrix was resolution enhanced in both dimensions by a cosine-bell function before Fourier Transformation. Solvent suppression was achieved with excitation sculpting sequence [29].

TOCSY experiment was acquired with a 70 ms mixing time. NOESY experiment was acquired with a 300 ms mixing time, and ROESY experiments with a 180 and 250 ms mixing time, using a continuous spin-lock. Heteronuclear single quantum coherence (1H, 13C HSQC) was performed using sensitivity improvement [30] and in the phase-sensitive mode using Echo/Antiecho gradient selection, with multiplicity editing during selection step.

According to Wüthrich [31], identification of amino-acid spin systems was performed by comparison of TOCSY and DQF-COSY, while sequential assignment was obtained by the analysis of NOESY and ROESY spectra (see S1 Table).

Data acquisition and processing were performed with Topspin 2.1 software package (Bruker).

### 2.8 Fluorescence imaging of dH3w-treated cell cultures

Human normal keratinocytes (HaCaT), were cultured in Dulbecco’s Modified Eagle’s Medium (DMEM), supplemented with 10% foetal bovine serum, 2 mM L-glutamine and 1% penicillin–streptomycin in a 5% CO2 humidified atmosphere at 37 °C.

Cells were seeded in 24-well plates (500 μL/well) on sterile cover-slips at a density of 5 x 10^4^/well and then grown at 37 °C for 48 hours. Cells were first treated with ZnCl (100 μM) for 3 hours. After incubation, three washes with PBS buffer were performed hence dH3w was added at the final concentration of 7 μM. After 2 hours, cells were incubated with HgCl_2_ (10 μM). Cells were analyzed under an Olympus BX51 fluorescence microscope (magnification 100X) using a DAPI filter (excitation 358 nm and emission 470 nm). Typical acquisition time was 500 ms. The images were captured using an Olympus DP70 digital camera (Olympus, NY, USA) and processed by using the analysis software supplied by the manufacturer.

## 3. Results and discussion

### 3.1. dH3w emission spectra in the presence of various concentrations of Hg^2+^

The fluorescence emission spectra of dH3w at pH 7 and increasing Hg^2+^ concentrations (up to 100 μM) were recorded after excitation at 340 nm, the maximum absorption wavelength of the dansyl moiety.

**Fig 1:**
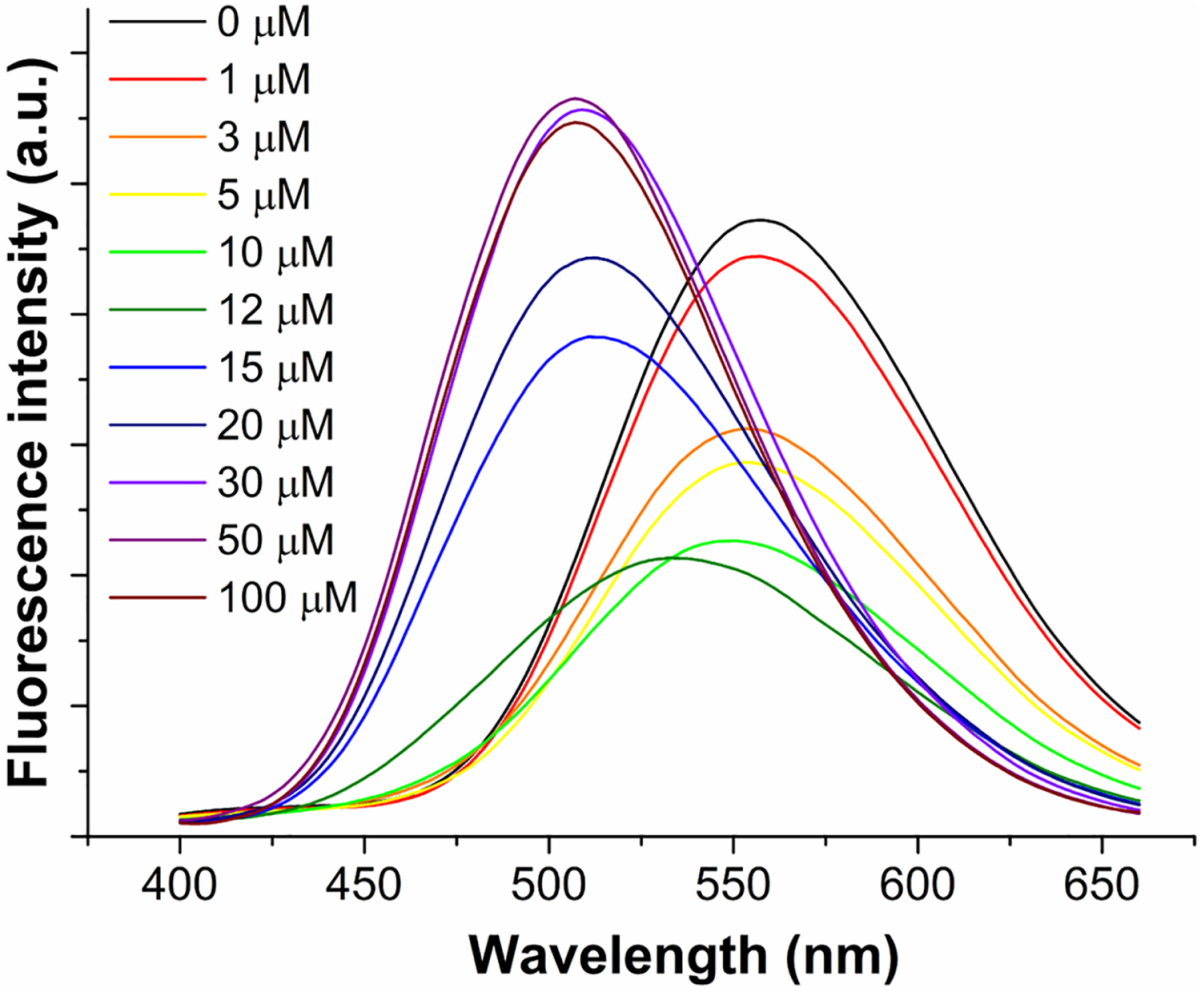
Fluorescence emission spectra of dH3w (7 μM) in the absence (black line) and in the presence of increasing concentrations of Hg^2+^ (1, 3, 5, 10, 12, 15, 20, 30, 50, 100 μM) at pH 7.0.

Unexpectedly two distinct behaviours were observed (Fig 1). At low Hg^2+^ concentrations (<10μM) dH3w showed a turn off response with a 60% reduction of the emission but no change in the spectrum features. At Hg^2+^ concentrations higher than 12 μM, dH3w showed a turn on response coupled with a noteworthy blue-shift of the λ_max_, from 560 to 510 nm.

This bimodal spectral behaviour strongly suggests the presence of at least two different binding modes of Hg ion having very different affinity constants.

**Fig 2:**
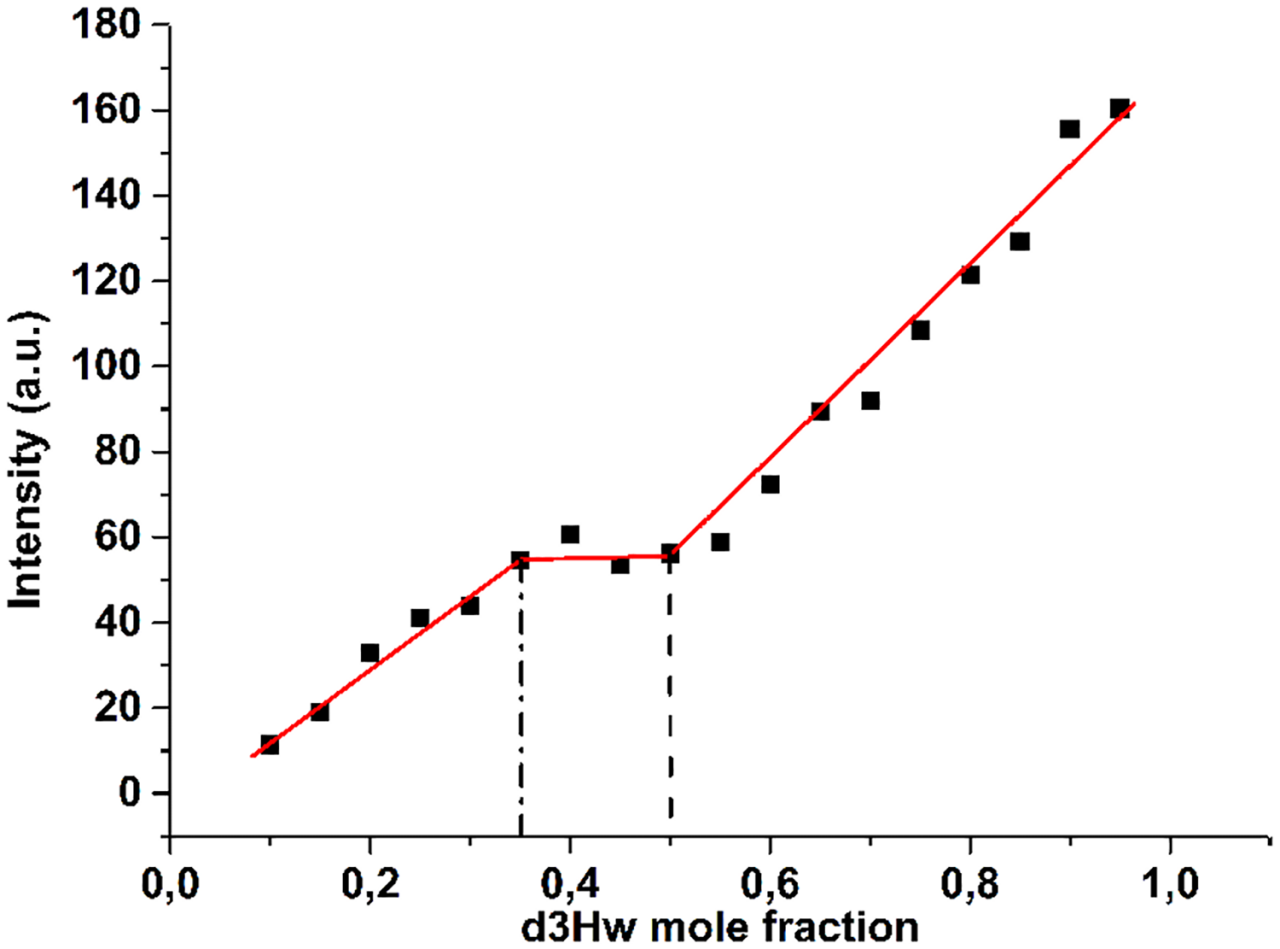
The Job’s plot obtained from the titration of dH3w peptide with Hg^2+^ ions. The dashed lines highlighted the found binding stoichiometry at mole fraction of dH3w of 0.52 and 0.34 which correspond to 1:1 and 1:2 (dH3w:Hg^2+^) complexes, respectively. The experiment was carried out in 20 mM Mops buffer, pH 7.0 at the temperature of 25 °C. Errors are not shown because they were always smaller than the symbols sizes.

To get more information on the complex binding behaviour of Hg^2+^ to dH3w we employed the continuous variations method. The Job’s plot (Fig 2) showed two intersection points at dH3w mole fractions of about 0.52 and 0.34 corresponding to stoichiometric ratios (dH3w:Hg) of 1:1 and 1:2 thus confirming that dH3w has two non equivalent binding sites for Hg ions.

**Fig 3:**
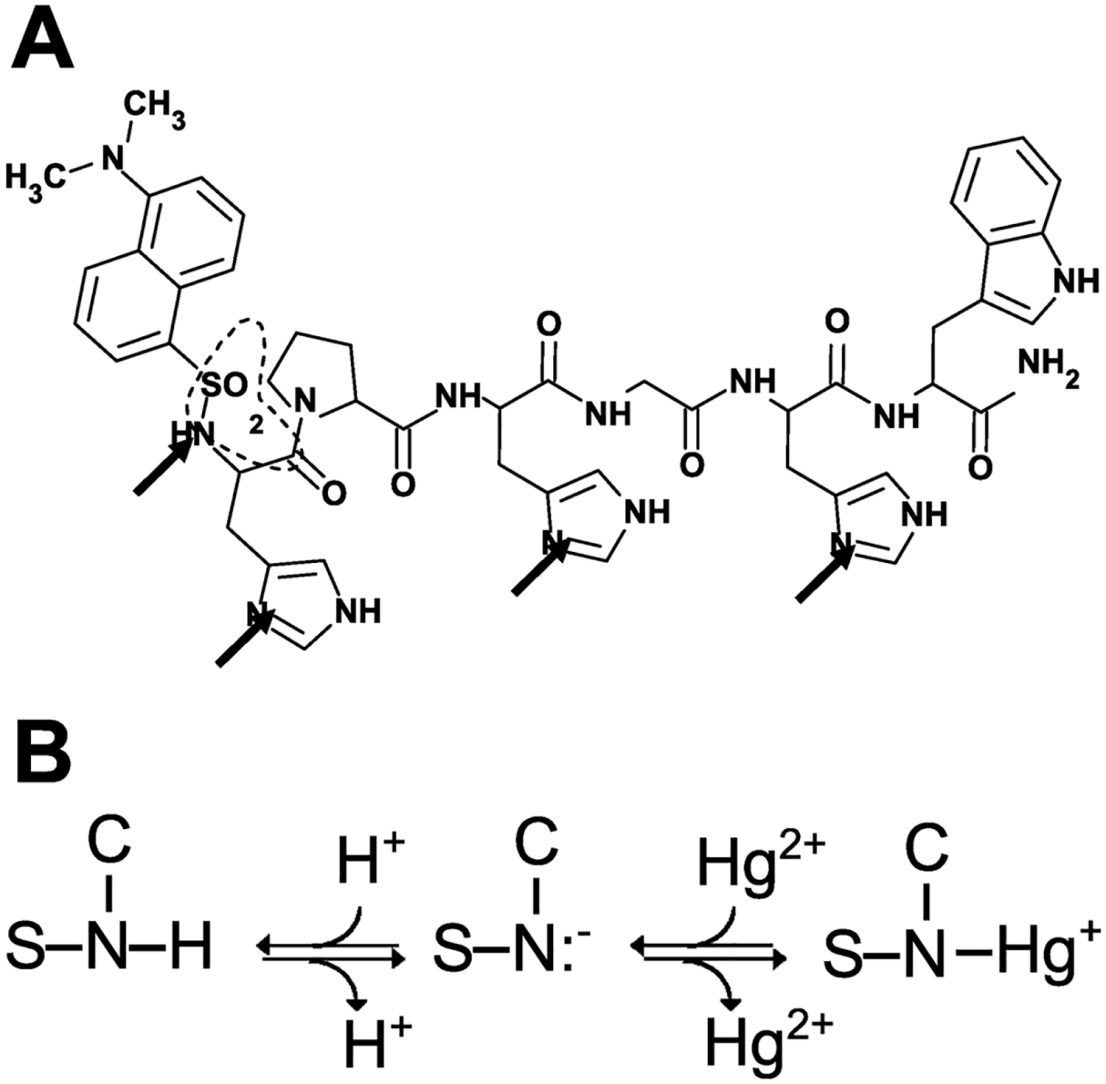
Structure of dH3w (A) and possible interaction mode of the sulphonamide moiety with Hg^2+^ (B). In panel A the arrows indicate the more likely Hg^2+^ ligands. Imidazole groups are arbitrarily shown with the proton at the Nε atom. The dashed line highlights the group shown in details in panel B.

dH3w contains at least four potential ligands for transition metal cations, i.e. the three imidazole moieties of the histidine residues at positions 1, 3 and 5 and the sulphonamide of the dansyl-sulphonamide fluorophore (Fig 3A). Other potential but lower affinity sites are the C-terminal amide and the internal peptide bonds. The significant blue-shift of the maximum emission observed at high Hg^2+^ concentrations can be likely attributed to the deprotonation of the dansyl-sulphonamide moiety upon binding to the strongly electrophilic Hg^2+^ ion. Direct binding of a dansyl-sulphonamide anion to Hg^2+^ has been previously suggested or demonstrated in several turn-on mercury sensors with different chemical structures [32]. In all these complexes, deprotonation and direct binding of the NH group of the sulphonamide to Hg^2+^ was always accompanied by a 40–50 nm blue shift in the emission due to the reduced conjugation between the deprotonated sulphonamide and the (dimethylamino)naphthalene moiety [32]. Moreover, it is well known that, in the presence of ligands with nitrogen as donor, Hg^2+^ prefers the (linear) 2-coordination and that a third ligand usually binds with a lower affinity [33–36]. Therefore, it is reasonable to hypothesize that, at low Hg^2+^ concentrations, a single Hg ion binds to the peptide through, likely, two or three histidine residues, whereas, at higher Hg^2+^ concentrations, a second ion binds through the sulphonamide (Fig 3B) and, possibly, one of the histidine residues.

To complete the characterization of the fluorescence properties of dH3w, we determined the quantum yield of dH3w in the absence and presence of saturating concentrations of Hg^2+^ (100 μM). The obtained values 0.049 ± 0.002 and 0.047 ± 0.005, respectively—show that Hg^2+^ binding does not change the quantum yield in spite of the large variations in the emission spectrum.

### 3.2. pH-titration curves in the presence of mercury(II)

As the pK_a_ of the imidazole and sulphonamide moieties are very different, about 6 and 10 respectively, in order to confirm the involvement of these groups in the binding process we studied the behaviour of the dH3w/Hg^2+^ complex as function of pH. The response of free dH3w to pH has been previously described [19]. In the pH range 4–8 the fluorescence emission of dH3w was scarcely influenced by pH [19]. At pH <4 the fluorescence intensity of dH3w decreased and disappeared completely below pH 2 likely due to protonation of the dimethylamino group, which prevents the charge transfer between the amine and naphthyl ring thus leading to the quenching of fluorescence. On the contrary at pH >8 the fluorescence intensity of dH3w increased and shifted of 50 nm toward the blue region due to the above-mentioned deprotonation of the sulphonamide moiety whose pK_a_ is ~ 10.

**Fig 4:**
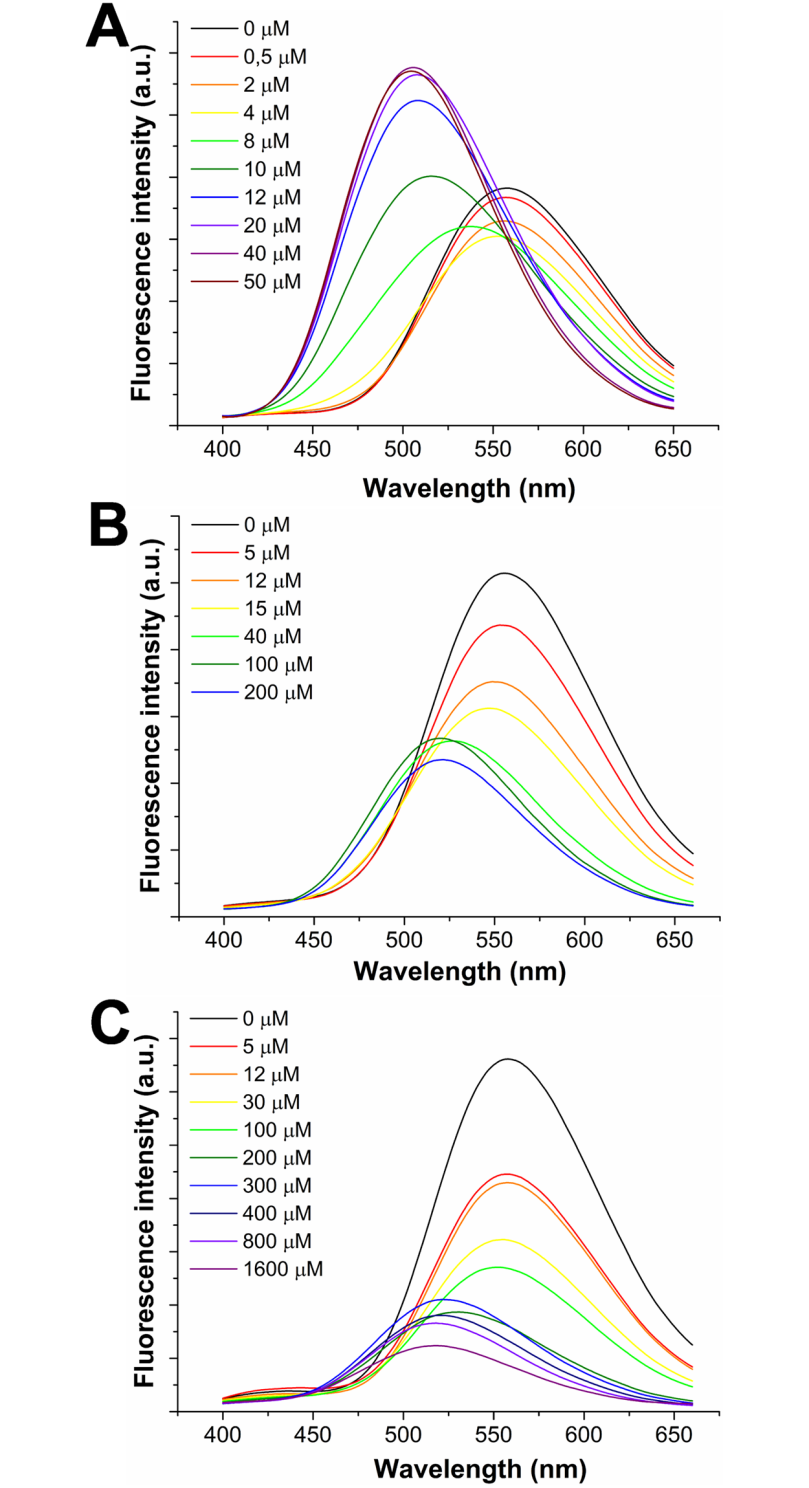
Fluorescence emission spectra recorded after excitation at 340 nm, of dH3w (7 μM) in the absence (black lines) and in the presence of increasing concentrations of Hg^2+^, at pH 8.0 (A), 6.0 (B) and 4.0 (C).

Therefore, we studied the interaction dH3w/Hg^2+^ at pH 8.0, 6.0 and 4.0. At all the pH values dH3w showed two distinct behaviours but with significant differences respect to pH 7. In particular, at pH 8 (Fig 4A) dH3w showed a turn off response only for Hg^2+^ concentrations lower than 8 μM and with a reduction of the emission of about 22%, i.e. much more modest with respect to that observed at pH 7. At higher concentrations, again a turn-on response and a blue-shift of the λ_max_, from 560 to 510 nm, was observed. Intriguingly at acidic pH values, high Hg^2+^ concentrations only induced the blue-shift not accompanied by the turn-on response (Fig 4B and 4C). Moreover, the blue-shift of the λ_max_ was observed at Hg^2+^ concentrations equal or higher than 40 μM at pH 6 (Fig 4B) and at concentrations equal or higher than 200 μM at pH 4 (Fig 4C). These findings are in agreement with the hypothesis that the second Hg^2+^ binding site includes a deprotonated dansyl-sulphonamide. At pH 7 the binding of the second Hg^2+^ can be described as a competition reaction between protons and Hg^2+^ (Fig 3B). As the concentration of H^+^ is held constant at 10^−7^ M by the buffer and the blue shift/turn-on response is observed at Hg^2+^ concentrations between 10^−5^ and 10^−4^ M it can be concluded that the affinity of Hg^2+^ for the sulphonamide group is 100–1000 times lower than that of the protons. At pH 8 the H^+^ concentration is ten times lower, therefore lower concentrations of Hg ions are needed to displace the proton from the sulphonamide nitrogen atom. The opposite is true at acidic pH were even the highest concentrations of Hg^2+^ tested can only partially replace protons in the NH group as evidenced by the blue shift in the of the λ_max_ not accompanied by the turn-on response. Obviously, acidic pH values should also weaken the binding of Hg^2+^ to histidine imidazole groups thus reducing also the binding constant of the first Hg^2+^. Unfortunately, the variations in the spectra as function of the Hg^2+^ concentration were too complex to perform a reliable fit allowing the direct determination of the two binding constants at the different pH values. The constants were instead determined at pH 7 by displacement of the Zn^2+^ ion from the dH3w/Zn^2+^ complex as described in the next section.

### 3.3 Interaction of dH3w/Zn^2+^ with Hg^2+^

**Fig 5:**
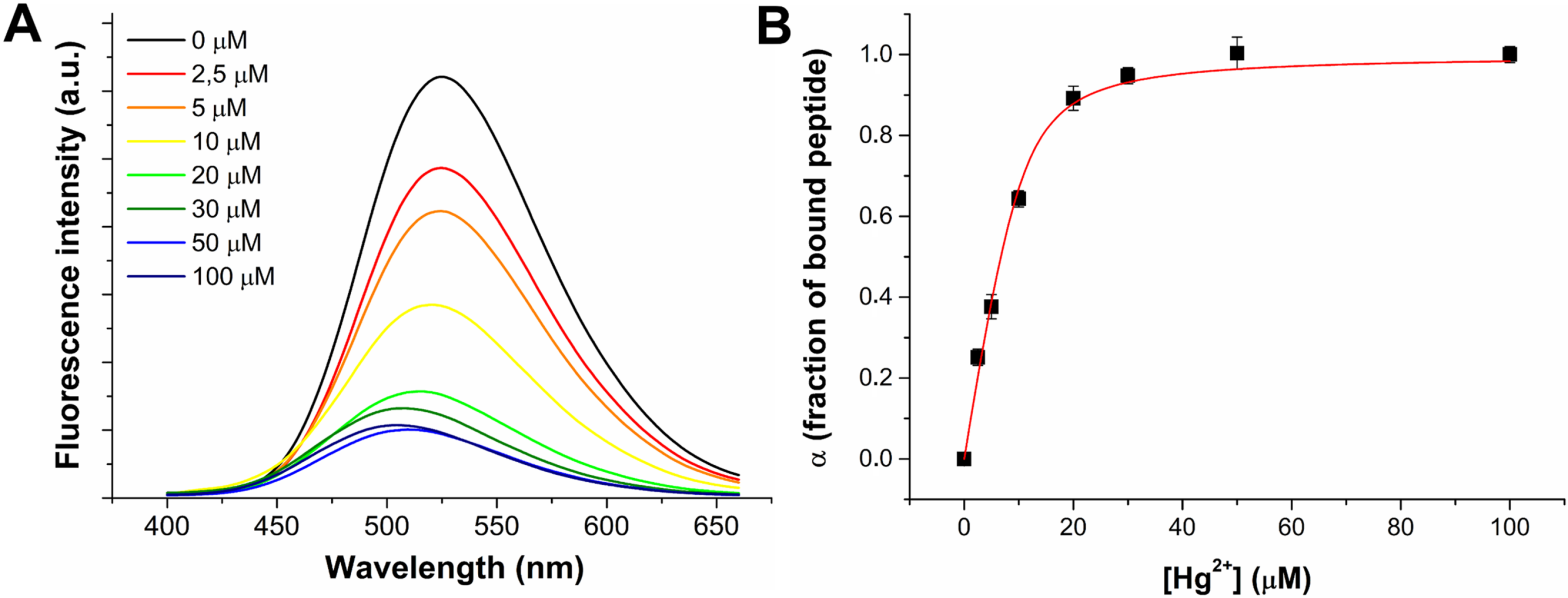
(A) Fluorescence emission spectra of Zn-complexed dH3w peptide (7 μM) in the presence of increasing concentration of Hg^2+^. The black line represents the spectrum of free peptide. (B) Binding curve obtained by plotting the fraction of bound peptide (α) versus the Hg^2+^ concentration. The red line represents the best fit of experimental points obtained using a two non-equivalent binding sites model. The experiment was carried out in 20 mM Mops buffer, pH 7.0 at the temperature of 25 °C.

We have previously shown that at pH 7 dH3w forms a stable complex with Zn^2+^ with a 2:1 stoichiometry and a binding constant of (5.9±2.2)∙10^5^ M^-1^ [19]. It is worth noting that zinc ion binding causes a strong turn-on response and fluorescence intensity increases 8–9 fold in the presence of saturating amounts of Zn^2+^. To evaluate whether Hg^2+^ is able to compete with Zn^2+^ for the binding to dH3w, we recorded the emission spectra of dH3w saturated with Zn^2+^ at increasing Hg^2+^ concentration in 20 mM Mops buffer, pH 7.0. Fig 5A shows fluorescence emission spectra obtained from the titration with Hg^2+^ of a 7 μM solution of dH3w peptide saturated with 200 μM of Zn^2+^. After the addition of Hg^2+^ to the Zn-complexed peptide, a decrease of fluorescence emission was observed together with a blue shift from ~525 nm (the λmax of the dH3w/Zn^2+^ complex) to ~509 nm suggesting that Hg^2+^ can bind dH3w displacing Zn^2+^. The binding curve, obtained by plotting the fraction of bound peptide (α) as a function of Hg^2+^ molarity, is shown in Fig 5B. Keeping in mind the results obtained for the titration in absence of Zn^2+^ (Figs 1 and 2), we modeled the binding curve by considering the presence of two independent and non-equivalent sites. This model accurately reproduces the experimental data providing apparent binding constants of 3∙10^7^ M^-1^ and 6∙10^5^ M^-1^. It should be highlighted that these constants are referred to the process in which an Hg^2+^ ion binds dH3w and simultaneously displaces a Zn^2+^. Considering the higher Zn^2+^ concentration used in these experiments, our results are consistent with Hg^2+^ binding constants for dH3w up to 1000-fold higher than the binding constant of Zn^2+^. Displacement of zinc ion was not studied at pH < 7 because at these pH values the interaction of dH3w with zinc is very weak or absent [19].

We have previously shown that Cu^2+^ is able to displace Zn^2+^ causing a turn off of the fluorescence of the dH3w/Zn^2+^ complex [19]. Therefore, in order to verify if other metal ions, in addition to Cu^2+^ and Hg^2+^, are able to displace Zn^2+^ from the dH3w/Zn^2+^complex, dH3w and Zn^2+^ were incubated in the presence of different concentrations of several heavy metal ions (Cu^2+^, Ni^2+^, Co^2+^, Mn^2+^, Pb^2+^, Cd^2+^, Fe^2+^, Sn^2+^ and Cr^3+^) and the fluorescence intensity at 515 nm was recorded. As shown in S3 Fig, except Cu^2+^, no other metal ion was able to quench the fluorescence of the dH3w/Zn^2+^ complex. It can be concluded that the dH3w/Zn^2+^complex is a turn-off sensor highly specific for copper and mercury.

### 3.4 NMR

In order to investigate the interaction of dH3w with Hg^2+^, NMR experiments were performed in H_2_O (10% D_2_O) by varying the metal-to-peptide ratio.

Upon addition of 1 equivalent of HgCl_2_ at pH 3.8, we observed a broadening of His^1^ imidazole protons and of Gly^2^, His^3^ and His^5^ amide protons. By increasing the pH to 4.5 the amide protons underwent a further broadening and this effect was also observed for almost all the aliphatic protons (see S4 Fig). The non-selective broadening of the above-mentioned protons, including those of non-coordinating groups (amide and aliphatic protons) could be ascribed to the presence of several coordination modes, which are in relatively slow (intermediate) exchange with each other and with the unbound peptide. No significant changes were observed by increasing the concentration of Hg^2+^ up to 20 equivalents (see S5 Fig), thus indicating a stoichiometric ratio Hg/dH3w of 1:1 at pH values in the range 3.8–4.5. This finding is in agreement with the results obtained studying the fluorescence of the dH3w in the presence of Hg^2+^ at acidic pH values (see section 3.2). Keeping in mind the above mentioned preference of Hg^2+^ for 2-coordination, it is likely to hypothesize that the dH3w/Hg^2+^ complex in solution exists as a mixture of three forms each with only two histidine residues bound to the metal ion (His^1^ + His^3^, His^1^ + His^5^ and His^3^ + His^5^). These three complexes could interconvert through a minor form with a tricoordinated Hg^2+^. Similar equilibria have been studied in the case of the interaction between histidine-rich peptides and immobilized divalent metal ions [37].

NMR experiments were also performed at pH 7.0 (50 mM phosphate buffer), in absence and in presence of HgCl_2_. During titration experiments with Hg^2+^ precipitate formation was observed and upon the addition of 1.0 equiv. of HgCl_2_, no NMR signal was recorded. This finding supports the formation of a dH3w-Hg(II) complex, that is poorly water-soluble at the concentration used in the NMR experiments (0.5 mM).

### 3.5 Detection limit

To assess the potential of dH3w as a probe for Hg^2+^, we determined the detection limit (LOD) at pH 4 and 7 in the absence of Zn^2+^ and at pH 7 in the presence of 100 μM Zn^2+^. To this aim we performed fluorescence titrations at low concentrations of Hg^2+^ (0–1200 nM). A good linear relationship between the fluorescence intensity at 540 nm and the metal ion concentration was obtained in all the conditions investigated (S6 Fig). The LOD values at pH 4 and 7 were 422±80 and 363±73 nM, respectively, whereas a LOD value of 56±12 nM was obtained at pH 7 in the presence 100 μM Zn^2+^, thus indicating that the dH3w/Zn^2+^ complex is a very sensitive probe for the detection of Hg^2+^.

### 3.6 Staining of HaCaT cells with dH3w and dH3w/Zn^2+^ to detect Hg^2+^

**Fig 6:**
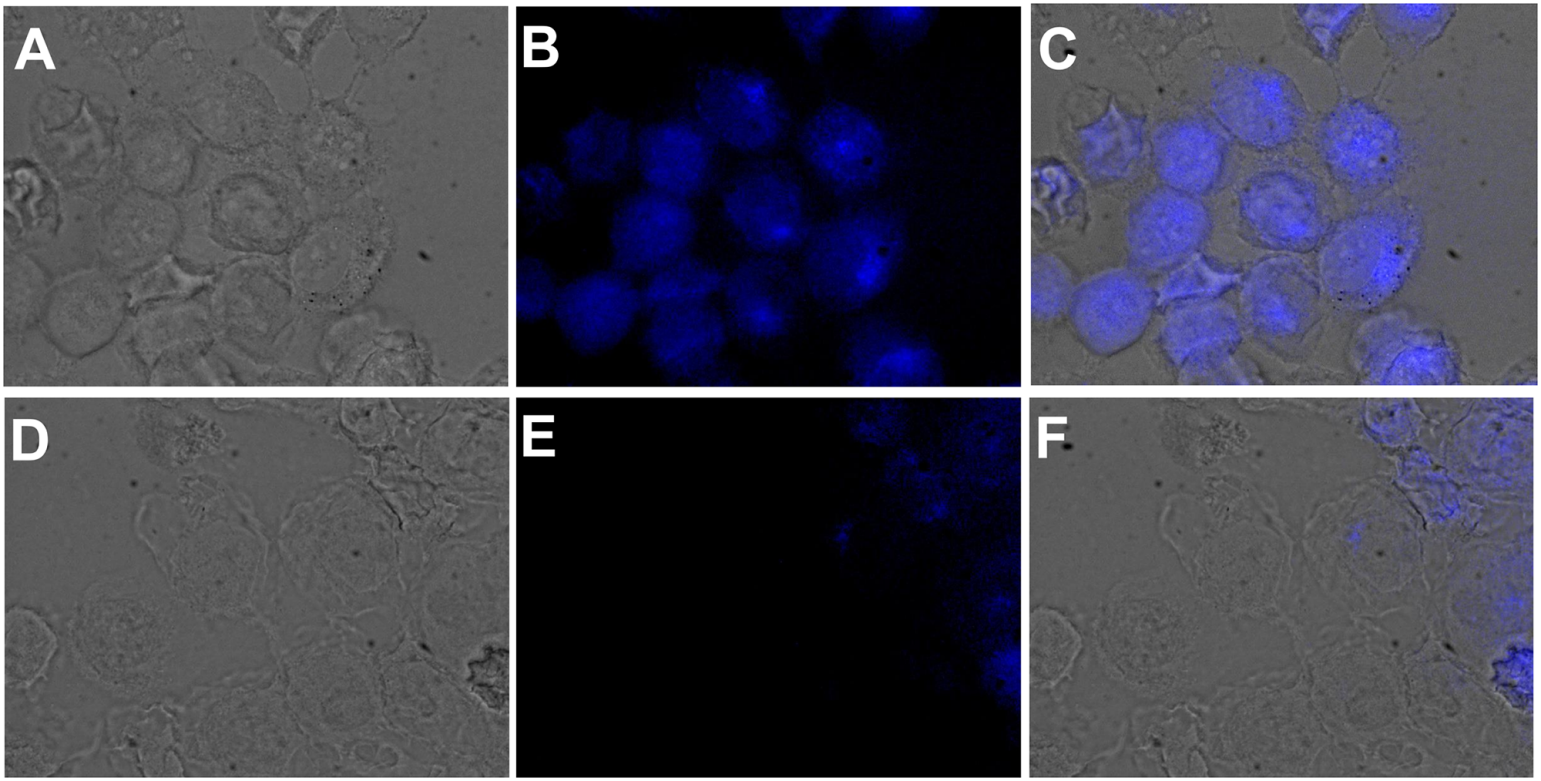
Fluorescent response of peptide to Zn^2+^ in the absence and presence of Hg^2+^ in HaCaT cells. A-C) Images of cells preincubated with Zn^2+^ for 3 h upon treatment with peptide. D-F) Images of cells after 2 h introduction of Hg^2+^. Conditions: [Zn^2+^] = 100 μM; [dH3w] = 10 μM; [Hg^2+^] = 10 μM. HaCaT cells were analysed by phase contrast (A,D) and fluorescence microscopy (B,E). Panels C and F are merged images.

AS we previously showed that dH3w is cell permeable [19] we also investigated the possible application of dH3w as a sensor for the detection of Hg^2+^ levels in eukaryotic cells. Preliminarily we determined the toxicity of dH3w and Zn^2+^ for HaCaT cells, a keratinocyte cell line from adult human skin. To this aim HaCaT cells were cultured in the presence of increasing concentration of dH3w and Zn^2+^ for 24 hours. As shown in S7 Fig, neither dH3W nor Zn^2+^ affected cell viability at any of the concentrations tested. HaCaT cells, after incubation with dH3w and Zn^2+^ (100 μM), showed a strong fluorescence observable both by using a blue and a green filter as expected from the spectral properties of the dH3w/Zn^2+^ complex described in the previous sections (Fig 6A–6C). After further exposure even to low amounts of Hg^2+^ (10 μM) the fluorescence was almost completely lost (Fig 6D–6F). These findings suggest that the dH3w/Zn^2+^ complex could also be used as probe to measure Hg^2+^ levels in cell cultures even if the necessity to excite the dansyl fluorophore in the UV region could limit its usefulness to short observation times.

## 4. Conclusions

We have shown that the fluorescent peptide dH3w binds two Hg^2+^ ions with different affinities. The first binding event determines a strong turn-off response and is relatively independent from pH being observed also at acidic pH values (pH 4–6). The second binding event likely involves the direct participation of the sulphonamide moiety of the fluorophore and is accompanied by relevant changes in the emission spectrum i.e. a blue shift of the λ_max_ and an increase of the emission intensity. As the binding of the sulphonamide to Hg^2+^ likely requires the deprotonation of its NH group, the second binding event is strongly dependent on the pH value and, at acidic pH values is observed only at very high concentrations of Hg^2+^. In spite of this complex behavior, at pH values in the range 4–7, dH3w is a convenient turn-off fluorescent sensor for the detection of Hg^2+^ in a 100% aqueous environment with a LOD of about 400 nm.

Moreover, Hg^2+^, like previously shown for Cu^2+^, easily displaces the zinc ion from the dH3w/Zn^2+^ complex. No other heavy metal ion is able to compete effectively with Zn^2+^ inducing the strong turn off response observed upon the addition of Hg^2+^ and Cu^2+^. Therefore, at neutral pH, the dH3w/Zn^2+^ complex can be used as a probe for the simultaneous selective detection of Hg^2+^ and Cu^2+^. It is worth noting that using dH3w/Zn^2+^ complex as a sensor the LOD for Hg^2+^ decreases to about 60 nM. As regards specificity and sensitivity dH3w is comparable to several other peptidyl fluorescent sensors [38–43]. This suggests that dH3w is suited both for environmental monitoring purposes in a wide range of pH values and for the detection of Hg^2+^ levels in cell cultures.

## Supporting information

S1 Fig dH3w HPLC analysis. (TIF)

S2 Fig dH3w MS spectrum. (TIF)

S3 Fig Fluorescence intensity changes of dH3W and Zn^2+^, in the presence of increasing concentrations of heavy metals. (TIF)

S4 Fig ^1^H NMR spectra in the amide and aliphatic regions of dH3w at pH 3.8 and 4.5. (TIF)

S5 Fig ^1^H NMR spectra in the amide region of dH3w at pH 4.5 in presence of different concentrations of Hg^2^. (TIF)

S6 Fig Detection limit (LOD) for Hg^2+^. (TIF)

S7 Fig Biocompatibility of the increasing concentrations of dH3W and Zn^2+^ on HaCaT cells. (TIF)

S1 Table Proton chemical shifts (δ, in ppm) of DH3W (C = 0.5 mM H_2_O/D_2_O 90/10) in absence and in presence of Hg^2+^. (PDF)
