# Supplementary figures and images for: Fluorescent peptide dH3w: A sensor for environmental monitoring of mercury (II)
Source: PLoS One. 2018 Oct 10;13(10):e0204164. doi: 10.1371/journal.pone.0204164 (PMC6179210; doi:10.1371/journal.pone.0204164)

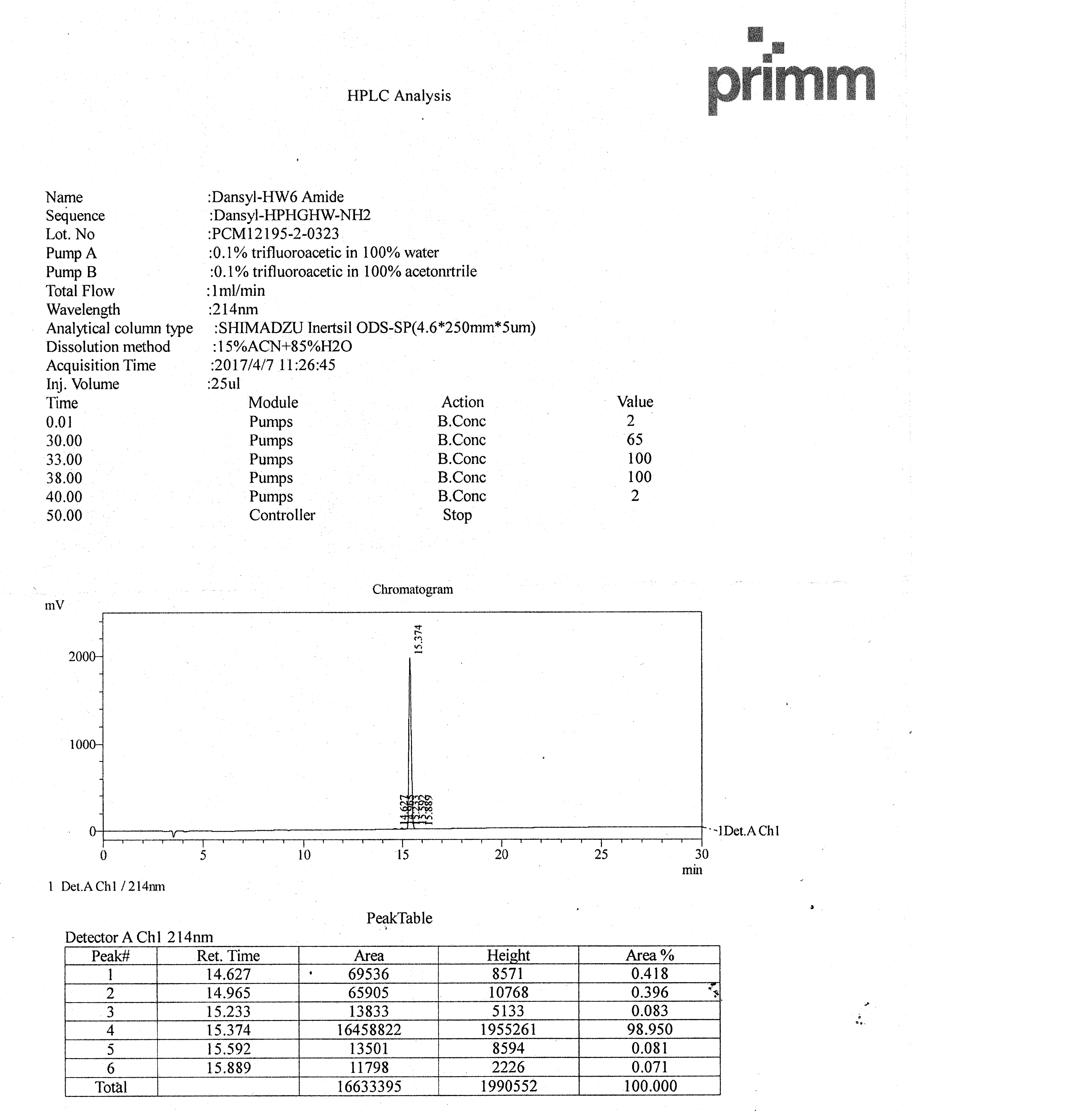

Supplement: S1 Fig — (TIF) [file pone.0204164.s001.tif]

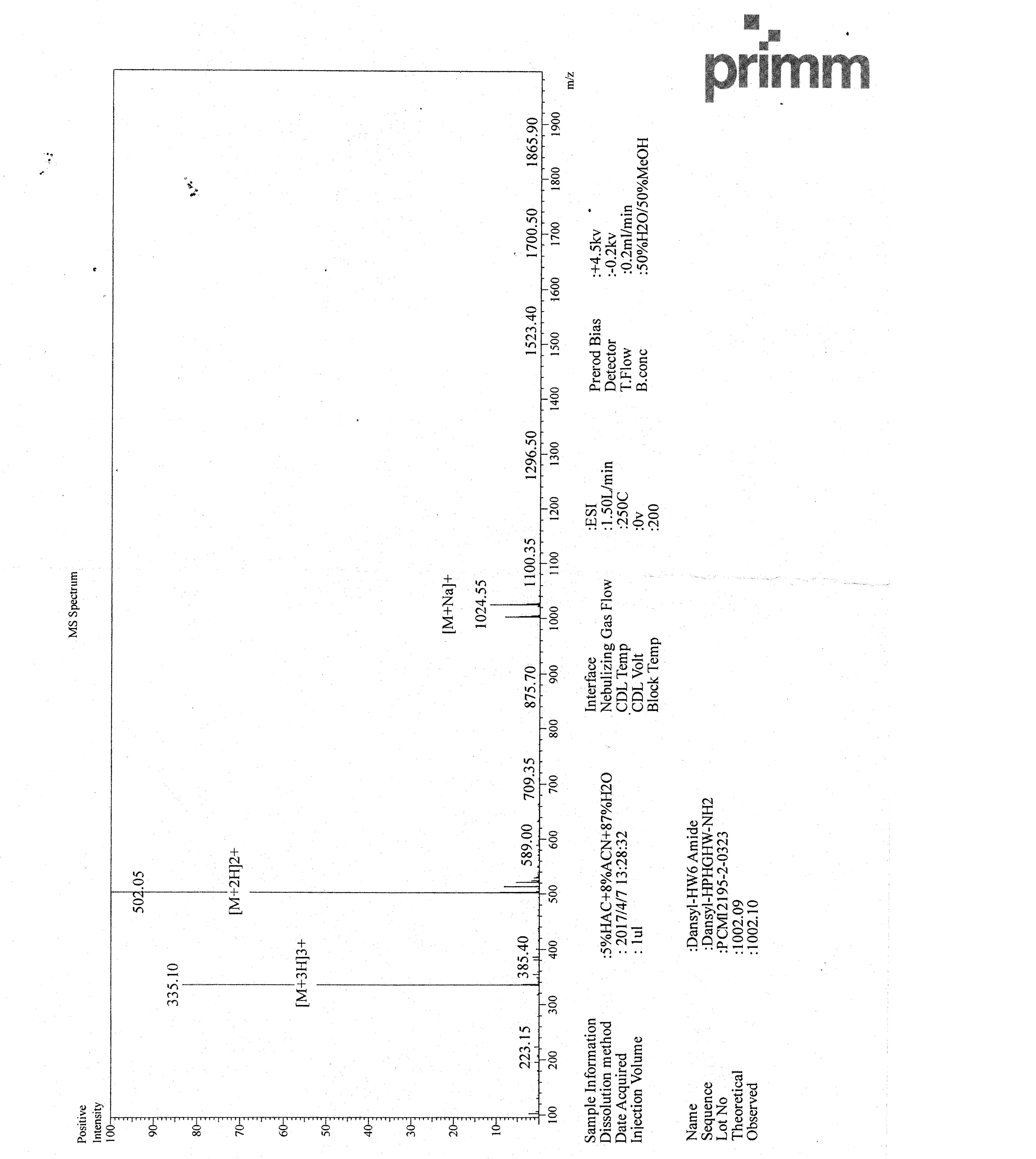

Supplement: S2 Fig — (TIF) [file pone.0204164.s002.tif]

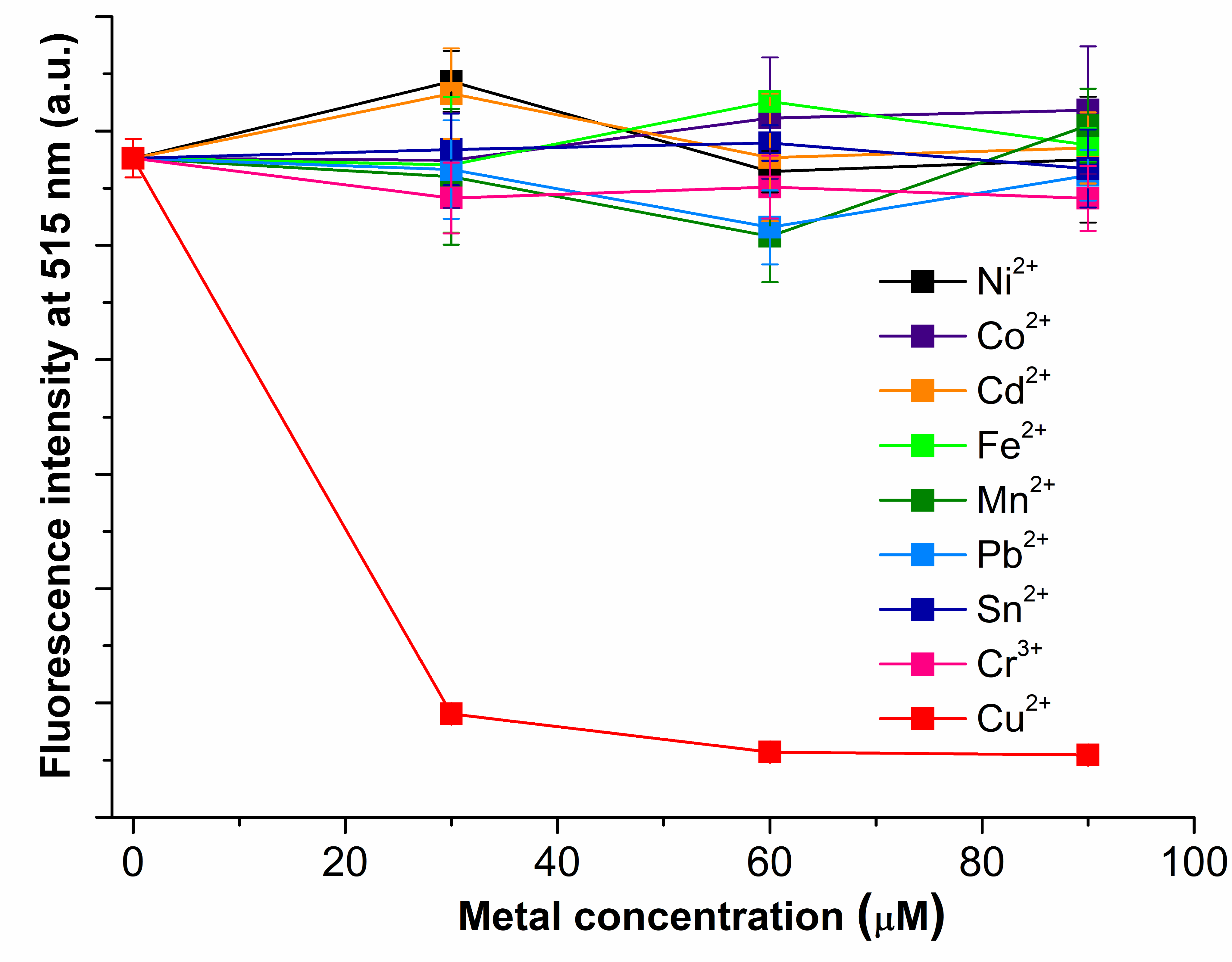

Supplement: S3 Fig — (TIF) [file pone.0204164.s003.tif]

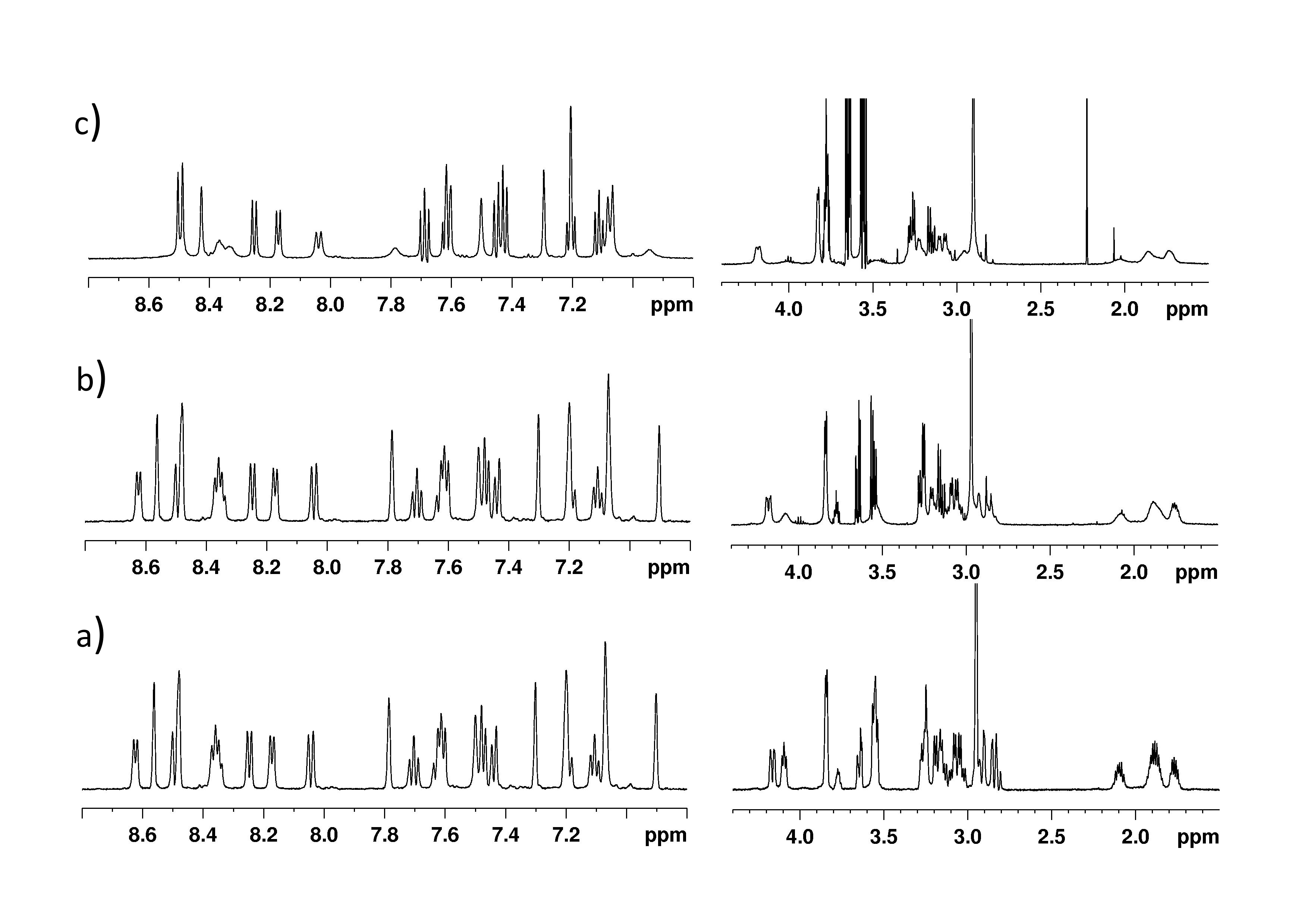

Supplement: S4 Fig — (TIF) [file pone.0204164.s004.tif]

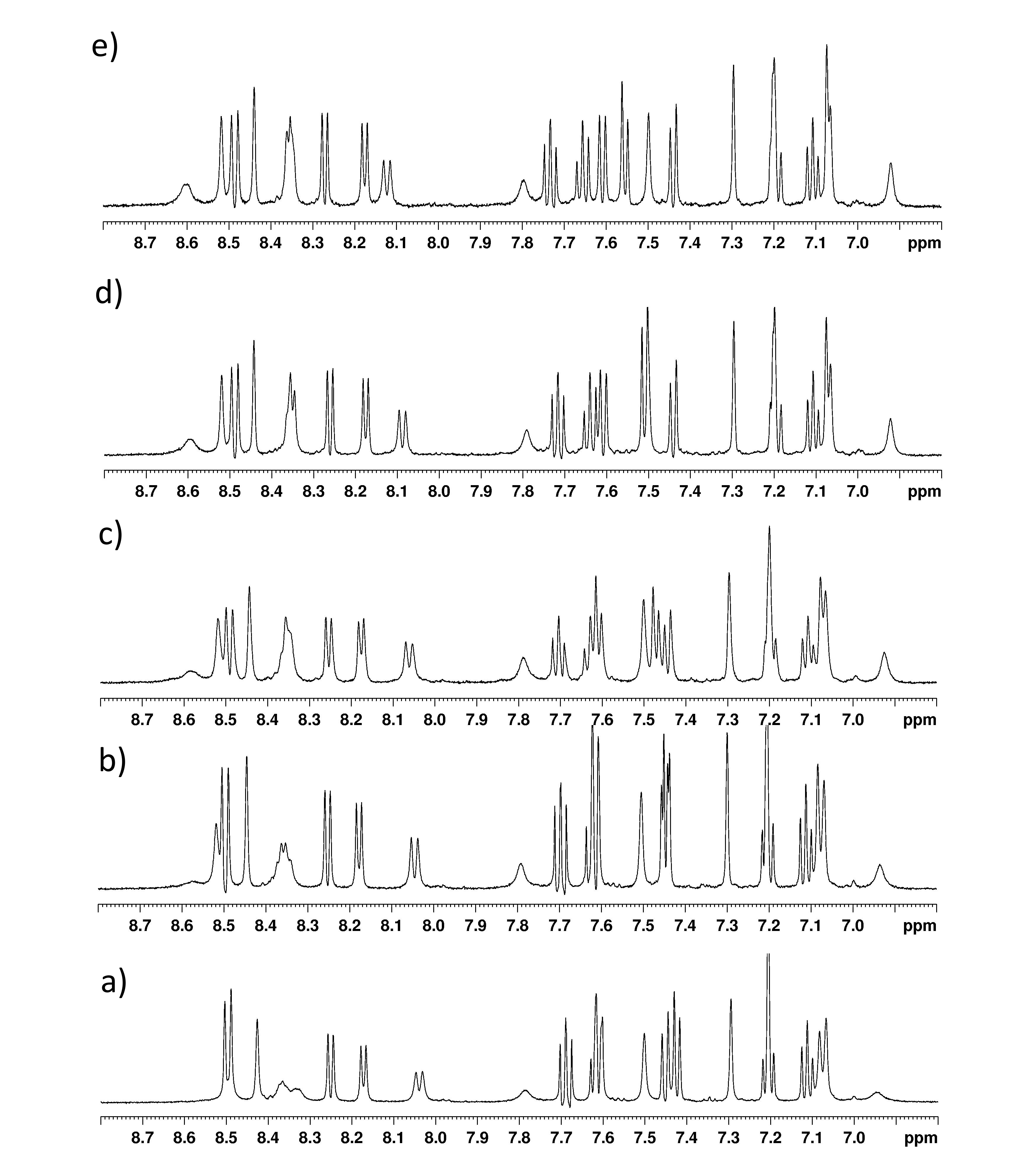

Supplement: S5 Fig — (TIF) [file pone.0204164.s005.tif]

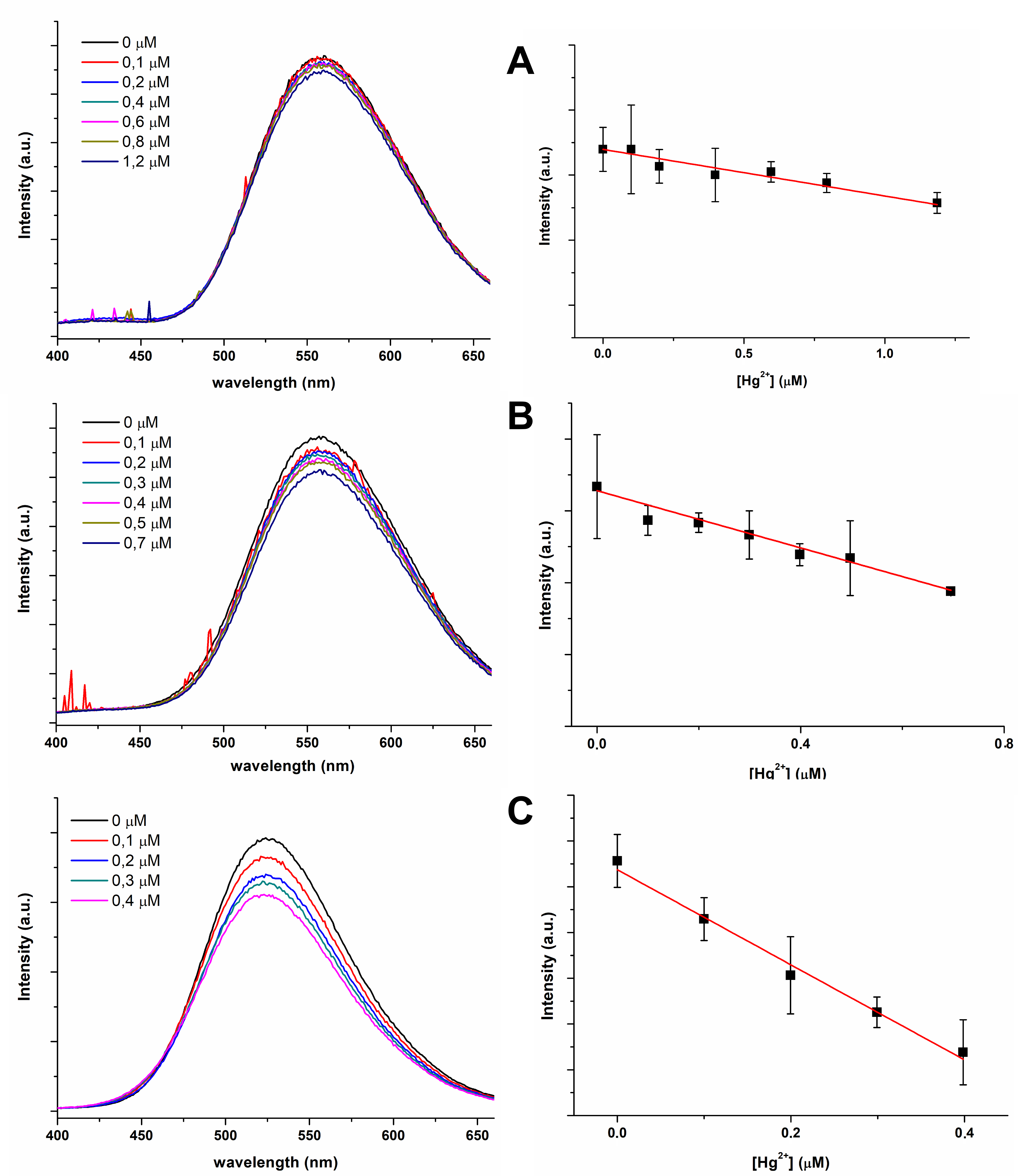

Supplement: S6 Fig — (TIF) [file pone.0204164.s006.tif]

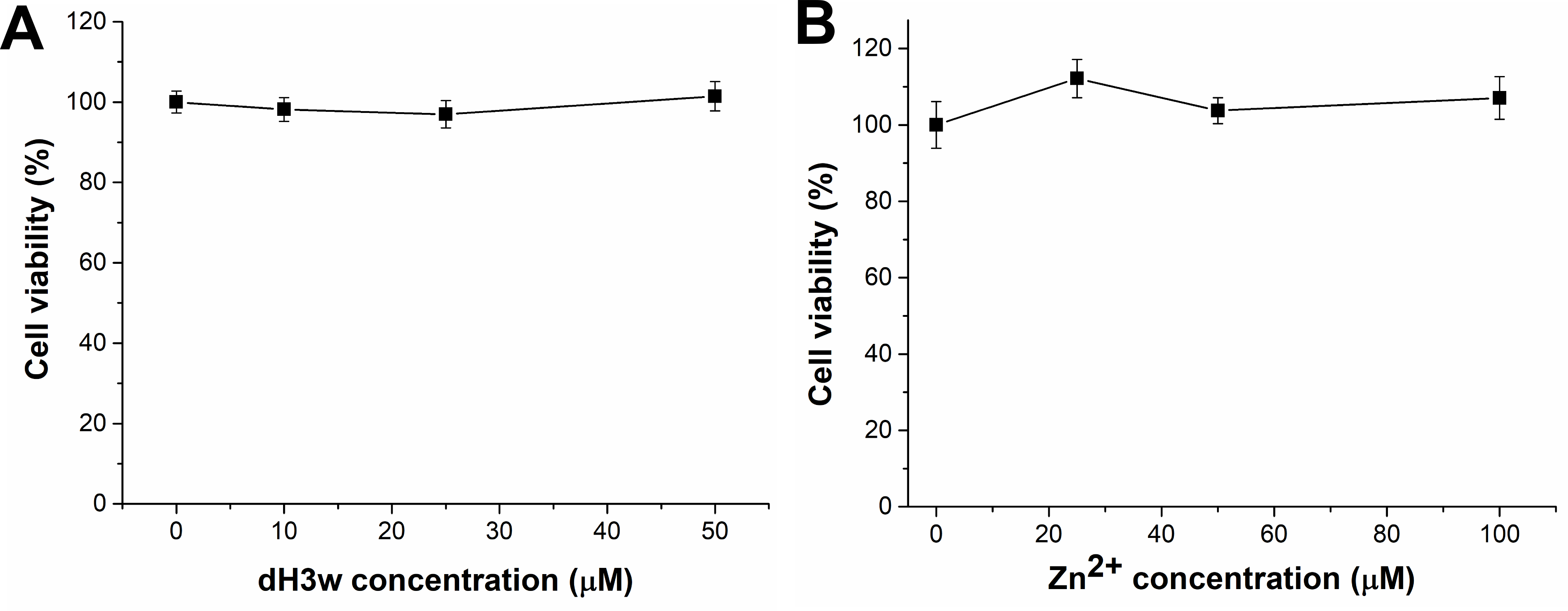

Supplement: S7 Fig — (TIF) [file pone.0204164.s007.tif]
