# Supplementary material for: Fluorescent peptide dH3w: A sensor for environmental monitoring of mercury (II)
Source: PLoS One. 2018 Oct 10;13(10):e0204164. doi: 10.1371/journal.pone.0204164 (PMC6179210; doi:10.1371/journal.pone.0204164)
Supplement: S1 Table — (PDF) [file pone.0204164.s008.pdf]

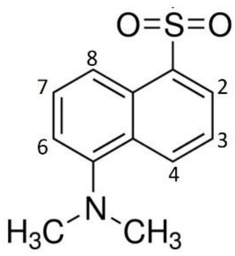

DH3W = Dansyl-His-Pro-His-Gly-His-Trp-NH_2

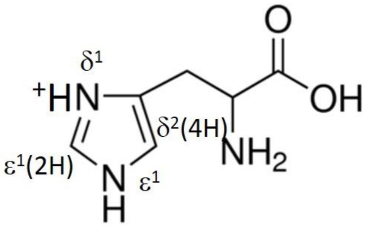

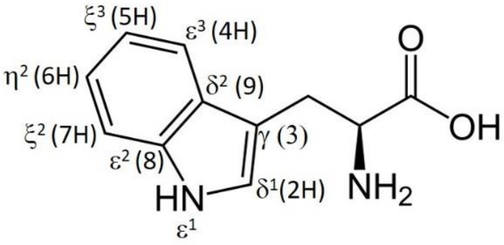

	DH3W	DH3W - Hg(II) (1eq. pH 3.8)	DH3W- Hg(II) (1eq. pH 4.5)
Dansyl			
**H2**	8.25	8.25	8.25
**H3**	7.72	7.71	7.69
**H4**	8.49	8.49	8.49
**H6**	7.47)	7.50	7.41
**H7**	7.61	7.63	7.62
**H8**	8.04	8.04	8.04
**CH_3**	2.94	2.93	2.93
His^{1}			
**NH**	-		
**αCH**	4.16		
**$\beta,\beta'$$CH_2$**	2.92 -2.84	2.92	2.92
**Hδ2 (4H)**	6.90	6.92	6.94
**Hε1 (2H)**	7.78	7.78	7.78
Pro^{2}			
**αCH**	4.09	4.03	4.03
**β,β' CH_2**	2.10 - 1.77	2.04 - 1.73	2.04 - 1.73
**γ,γ' CH_2**	1.88	1.86	1.86
**δ,δ' CH_2**	3.56	3.46	3.46
His^{3}			
**NH**	8.63	8.61	-
**αCH**	4.65	-	
**β,β' CH_2**	3.23 - 3.17	3.25	3.25
**Hδ2 (4H)**	7.30	7.30	7.30
**Hε1 (2H)**	8.56	8.54	8.48
Gly^{4}			

**NH**	8.35	8.37	8.37
**α,α' CH_2**	3.84	3.83	3.83
His^5			
**NH**	8.37	8.35	8.35
**αCH**	4.59	4.58	4.58
**β,β' CH_2**	3.06	3.10	3.10
**Hδ2 (4H)**	7.07	7.08	7.08
**Hε1 (2H)**	8.47	8.46	8.43
Trp^6			
**NH**	8.17	8.17	8.17
**αCH**	4.60	4.62	4.62
**β,β' CH_2**	3.26 - 3.14	3.27 - 3.17	3.27 - 3.17
**Hδ1 (2H)**	7.18	7.21	7.21
**Hε1 (NH)**	10.09	10.09	10.09
**Hε3 (4H)**	7.60	7.60	7.60
**Hξ3 (5H)**	7.11	7.11	7.11
**Hη2 (6H)**	7.20	7.20	7.20
**Hξ2 (7H)**	7.44	7.44	7.44
NH_2			
**H11**	7.50	7.50	7.50
**H12**	7.07	7.07	7.07

S3 Table. Proton chemical shifts (δ, in ppm) of DH3W (C = 0.5 mM H_2O/D_2O 90/10) in absence (pH = 3.8) and in presence of Hg^{2+}, (pH = 3.8 and pH = 4.5)
